# Ion Channel Modeling beyond State of the Art: A Comparison with a System Theory-Based Model of the Shaker-Related Voltage-Gated Potassium Channel Kv1.1

**DOI:** 10.3390/cells11020239

**Published:** 2022-01-11

**Authors:** Sonja Langthaler, Jasmina Lozanović Šajić, Theresa Rienmüller, Seth H. Weinberg, Christian Baumgartner

**Affiliations:** 1 Institute of Health Care Engineering with European Testing Center for Medical Devices, Graz University of Technology, A-8010 Graz, Austria; s.langthaler@tugraz.at (S.L.); j.lozanovicsajic@tugraz.at (J.L.Š.); theresa.rienmueller@tugraz.at (T.R.); 2 Innovation Center of the Faculty of Mechanical Engineering, University of Belgrade, 11000 Belgrade, Serbia; 3 Department of Biomedical Engineering, The Ohio State University, Columbus, OH 43210, USA; weinberg.147@osu.edu; 4 Davis Heart and Lung Research Institute, The Ohio State University Wexner Medical Center, Columbus, OH 43081, USA

**Keywords:** ion channels, electrophysiology, computational model, Hodgkin–Huxley, hidden Markov model, system and control theory

## Abstract

The mathematical modeling of ion channel kinetics is an important tool for studying the electrophysiological mechanisms of the nerves, heart, or cancer, from a single cell to an organ. Common approaches use either a Hodgkin–Huxley (HH) or a hidden Markov model (HMM) description, depending on the level of detail of the functionality and structural changes of the underlying channel gating, and taking into account the computational effort for model simulations. Here, we introduce for the first time a novel system theory-based approach for ion channel modeling based on the concept of transfer function characterization, without a priori knowledge of the biological system, using patch clamp measurements. Using the shaker-related voltage-gated potassium channel Kv1.1 (KCNA1) as an example, we compare the established approaches, HH and HMM, with the system theory-based concept in terms of model accuracy, computational effort, the degree of electrophysiological interpretability, and methodological limitations. This highly data-driven modeling concept offers a new opportunity for the phenomenological kinetic modeling of ion channels, exhibiting exceptional accuracy and computational efficiency compared to the conventional methods. The method has a high potential to further improve the quality and computational performance of complex cell and organ model simulations, and could provide a valuable new tool in the field of next-generation in silico electrophysiology.

## 1. Introduction

Mathematical models of individual ion channels form the building blocks of electrophysiological in silico approaches, allowing the investigation of biophysical mechanisms and the bioelectric activity of excitable and non-excitable cells [1,2]. A variety of whole-cell models of different levels of complexity and abstraction have been introduced for the simulation of ion current kinetics and action potential alterations in neural and cardiac cells, facilitating the prediction of disease processes and the development of therapeutic interventions, which have become an integral part in neuroscience and cardiac electrophysiology [1,3,4,5,6,7]. Furthermore, single-channel models predicting emergent ion channel drug effects on both cellular and tissue levels are increasingly under consideration in pharmacological research, in conjunction with experimental investigations, opening up an innovative and efficient way of early preclinical drug screenings [4]. Hence, a maximum possible degree of biophysical detail with a simultaneously acceptable computational burden is fundamental and represents a main challenge for the reliable and successful integration and application of ion channel models in biomedical research.

The mathematical modeling of channel kinetics is commonly based on either a Hodgkin–Huxley (HH) or a hidden Markov model (HMM) description [1,8,9,10,11]. The HH model offers a basic paradigm in which the channel can be either open or closed depending on a set of gates, controlled by a number of gating particles, representing the activation, deactivation, and inactivation characteristics of the ion channel type. The kinetic behavior of each gating particle between a permissive and non-permissive state is described as a first-order process, independent of all states of the other gates, and, thus, does not consider the possible dependences between the activation and inactivation of the channel [9,10,12]. However, although these models lack the underlying electrophysiological processes of channel gating, HH models closely reproduce the macroscopic currents with a small number of variables and a low computational burden and are, hence, still widely used in computational electrophysiology [1,11].

Hidden Markov models, by comparison, specify channel states according to the protein conformation and, thus, take into account the channel-specific gating behavior, enabling a highly accurate and veritable modeling of the channel kinetics [10,11,12,13]. Since the opening of individual ion channels is a stochastic process that can be described as a series of dependent transitions between distinct conformational states, the Markov schemes offer an optimal framework for modeling the microscopic current of single ion channels [10,11]. In particular, the investigation of channelopathies or drug-specific effects on the gating behavior through targeted changes in certain conformational states requires the use of such a probabilistic method, where, ideally, each state would correspond to one protein conformation [11,14]. In practice, however, even complex Markov models are only approximations to the actual channel dynamics [11]. Nevertheless, the high level of detail results in a huge number of parameters, increasing the computational cost for both the parametrization and simulation, while also increasing the risk of overfitting. Hence, various simplifications and models with reduced numbers of states are proposed in order to keep the computational burden as low as possible, while sufficiently depicting the complex protein structure and modeling the measured ion current. Such simplified models are phenomenological rather than representing the actual conformational states and are generally used in a way, similar to HH models, to simulate the measured macroscopic currents from whole-cell measurements deterministically [1,11].

In this work, using the shaker-related voltage-gated potassium channel Kv1.1 (KCNA1) as an example, a novel system theory-based (STB) approach for ion channel modeling is presented based on the concept of transfer function characterization using experimental data from patch clamp experiments, with voltage protocols for channel stimulation and measured macroscopic currents as input- and output information of the system. The developed system theory-based concept is compared with the two established approaches, the HH model and HMM, in terms of (i) the accuracy of model simulations, (ii) the computational effort of building models and running simulations, (iii) the degree and level of electrophysiological interpretability, and (iv) the methodological limitations. For the evaluation and verification of the models, data from patch clamp measurements of CHO cells are used, stably expressing rat Kv1.1 channels at a physiological temperature, obtained from the ion channel knowledge base Channelpedia [15].

This strongly data-driven modeling concept provides a new method for the phenomenological kinetic modeling of ion channels without a priori knowledge of the biological system, with an exceptional model accuracy and computational efficiency compared to the state-of-the-art methods, which is urgently needed in view of the development of increasingly complex cell and organ models.

## 2. Methods and Results

### 2.1. Electrophysiological Experiments and Datasets

**Figure 1:**
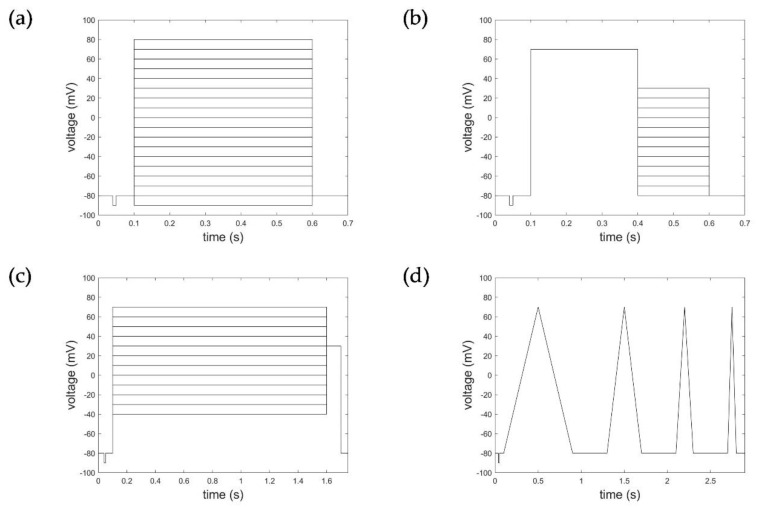
Voltage step and ramp protocols. (**a**) Activation protocol, (**b**) deactivation protocol, (**c**) inactivation protocol, and (**d**) ramp protocol.

Comprehensive experimental data on Kv1.1 channels were provided via the ion channel knowledge base Channelpedia (https://channelpedia.epfl.ch, accessed on 16 November 2021) [15]. Data used for model evaluation were based on the CHO_FT Rat KV1.1 35 °C dataset containing 66 individual cell measurements for activation protocols and 54 individual cell measurements for deactivation, inactivation, and ramp protocols (see Figure 1). CHO cells (Chinese hamster ovarian cells), stably expressing rat Kv1.1 channels without Kvβ1 and Kvβ2 subunit expression, were used for measurement of the Kv1.1 macroscopic currents. Electrophysiological recordings were performed with the automated patch clamp system Nanion NPC-16 Patchliner Quattro (Nanion Technologies, Munich, Germany), equipped with EPC-10 HEKA Quadro amplifiers (HEKA Elektronik, Reutlingen, Germany), PatchControlHT software (Nanion Technologies, Munich, Germany), and temperature control in whole-cell configuration. Basic quality criteria for measurements were met, showing an offset voltage of V_offset_ < 45 mV, seal resistance of R_seal_ > 200 MΩ (after whole-cell configuration), series resistance of R_series_ < 15.5 MΩ, and membrane capacitance of C_slow_ < 35 pF. Data were further processed based on calculated activation index (AI), maximum currents, and a subsequent manual exclusion of measurements [15].

Macroscopic currents were recorded to activation protocols consisting of a 100 ms long initial- and re-pulse at −80 mV and pulses starting from −90 mV to 80 mV (in increments of 10 mV) of 500 ms duration (Figure 1a). The deactivation protocol applied consisted of an initial- and re-pulse of −80 mV for 100 ms, a depolarization pulse at 70 mV over 300 ms for activation, followed by 300 ms long deactivation pulses from −80 mV to +30 mV in 10 mV steps (Figure 1b). Inactivation characteristics were measured according to a voltage protocol of an initial- and re-pulse of −80 mV for 100 ms, depolarization pulses from −40 mV to 70 mV (increment 10 mV) of 1500 ms duration, followed by an activation pulse of 30 mV for 100 ms (Figure 1c). The ramp protocols considered comprised four intervals of de- and hyperpolarization ranging from −90 mV to 50 mV with varying pulse duration (400 ms, 200 ms, 100 ms, 50 ms) and 400 ms pulse breaks to allow the channels to recover (Figure 1d).

### 2.2. Data and Data Pre-Processing Considered for HMM and STB Model Parameterization

Model parameterization was based on pre-processed data, excluding cell measurements with seal resistance R_seal_ < 300 MΩ and cell measurements exhibiting a high noise level or seal instabilities, resulting in a sample size of *n* = 60 cells for the activation curves, *n* = 37 cells for the deactivation curves, *n* = 45 cells for inactivation curves, and *n* = 54 cells for the ramp curves. The measured voltage steps considered for parametrization of the HMM model were limited from −50 mV to 70 mV for the activation protocol and from −80 mV to −30 mV for the deactivation protocol, representing voltage levels at which deactivation occurs after channel activation.

### 2.3. Available HH Model and HMM of the Ion Channel Kv1.1

Several HH models [15,16] and HMM-based approaches were developed for the Kv1.1 ion channel family, modeling their native gating behavior as well as specific ion channel–drug interactions, such as the effect of fluoxetine or syntaxin on channel activation [17,18,19,20,21,22]. The channels were reported as non- or slowly inactivating at room temperature, but exhibited a fast inactivation when co-expressed with Kvβ1 or Kvβ3 subunits [23,24,25,26]. A comparably strong inactivation was similarly observed near physiological temperature even in the absence of β subunits [15].

As current HMMs only reflect the activation behavior of these channels at room temperature, while a possible inactivation is not or only insufficiently considered in the proposed Markov schemes, the currently available HMM approaches can, as a consequence, scarcely be adopted and applied for the simulation of other datasets, in particular at higher or physiological temperature levels. Hence, in order to subsequently provide a reliable juxtaposition of the different modeling approaches, here, we further developed an HMM for simulating the macroscopic current of Kv1.1, that also takes into account the slow and fast inactivation at physiological temperature.

### 2.4. Mathematical Concepts of Ion Channel Modelling

#### 2.4.1. The System Theory-Based Modeling Approach for the Kv1.1 Channel

**Figure 2:**
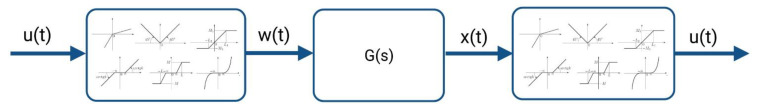
The Hammerstein–Wiener model.

In contrast to traditional modeling concepts in computational electrophysiology such as Hodgkin–Huxley or hidden Markov-based models, system identification, a methodology known from the field of control engineering and system theory, deals with the characterization of linear or non-linear systems based on observed input and output data. This approach involves specification of the model structure, estimation of the unknown model parameters, and validation of the resulting model. As the kinetics of an ion channel can be considered as a non-linear system in which the output information is not proportional to the change in the input information, we pursued a non-linear system identification approach for modeling. After a detailed analysis of the measured Kv1.1 macroscopic currents to the given input voltage protocol, the Hammerstein–Wiener (HW) model, which is a block-structured system model, was selected. The HW model consists of a linear dynamic subsystem *G*(*s*) between two static nonlinear elements, as shown in Figure 2 [27,28].

**Figure 3:**
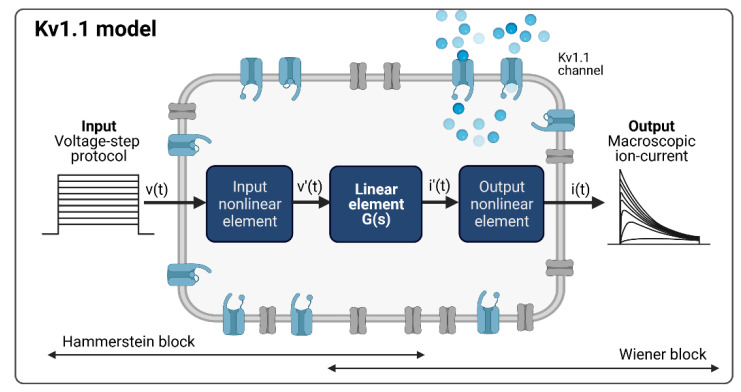
Kv1.1 cell model based on the Hammerstein–Wiener model. Created with BioRender.

Considering the common patch clamp recordings with voltage step and ramp protocols as system input functions and the measured macroscopic current as the system output function, the Kv1.1 channel model, according to the Hammerstein–Wiener model structure, is shown in Figure 3, with the measured activation curves from a voltage step protocol as an example.

The Kv1.1 model with input *v*(*t*) and output *i*(*t*) nonlinearities of the HW-based Kv1.1 ion channel model was structured as piecewise linear (*v*′(*t*) and *i′*(*t*)) with two breakpoints between input and output nonlinearities. Note that input and output nonlinearities can also be defined as a sigmoid network, piecewise linear with more breakpoints, saturation, dead zone, wavelet network, one-dimensional polynomial, or other elements known from control engineering. Here, we considered different input and output nonlinearities and adopted a model with the same type of input and output nonlinearities.

In general, the HW-based Kv1.1 ion channel model can be described as:

(1) $$output\left(t\right)=\{\begin{array}{c} k_{1}\cdot input\left(t\right),\;\;input\left(t\right)\epsilon \left(0,t_{1}\right)\\ \begin{array}{c} k_{2}\cdot input\left(t\right),\;\;input\left(t\right)\epsilon \left(t_{1},t_{2}\right)\\ k_{3}\cdot input\left(t\right),\;\;input\left(t\right)\epsilon \left(t_{2},t_{3}\right) \end{array} \end{array}$$

For Kv1.1, the system input is defined as *v*(*t*), i.e., the voltage signal according to the applied protocol, and system output is *i*(*t*), i.e., the measured macroscopic current. According to the definition of the block-structured HW model, it is necessary to define intermediate input functions, *v_i_*(*t*), and intermediate output functions *i_i_*(*t*). The intermediate input is the output of the input nonlinear element and the input to the linear element *G*(*s*). Analogously, the intermediate output is the output of the linear element *G*(*s*) and the input of the output nonlinear element. The intermediate input and the output functions are defined in Equation (2) and Equation (3), respectively.

(2) $$v^{\prime }\left(t\right)=\{\begin{array}{c} k_{1}\cdot v\left(t\right),\;\;\\ \begin{array}{c} k_{2}\cdot v\left(t\right),\;\;\\ k_{3}\cdot v\left(t\right)\;\; \end{array} \end{array}$$

(3) $$i\left(t\right)=\{\begin{array}{c} l_{1}\cdot i^{\prime }\left(t\right),\;\;\\ \begin{array}{c} l_{2}\cdot i^{\prime }\left(t\right),\;\;\\ l_{3}\cdot i^{\prime }\left(t\right) \end{array} \end{array}$$

The linear element *G*(*s*) is the transfer function (TF), which represents the differential equation of the dynamic behavior of the system. The TF is a mathematical representation between an intermediate input and an intermediate output function of the system. Hence, the TF of a linear system is defined as the ratio of the Laplace transform of the output to the Laplace transform of the input, where all initial conditions are assumed to be equal to zero (see Equation (4)) [29,30].

(4) $$G\left(s\right)=\frac{ℒ\left\{output\left(t\right)\right\}}{ℒ\left\{input\left(t\right)\right\}}=\frac{Output\left(s\right)}{Input\left(s\right)}$$

According to Figure 3, the system output is represented by the measured Kv1.1 macroscopic current *i*(*t*) in the time domain, with its equivalent in the s- or Laplace-domain, the function *I*(*s*), where *I*(*s*) is the Laplace transform of *i*(*t*), *I*(*s*) = ℒ{*i*(*t*)}. The system input function is an arbitrary function in the time domain according to the applied voltage step protocol used for stimulation of the Kv1.1 channel, and is defined as *v*(*t*) in Laplace domain as *V*(*s*) = ℒ{*v*(*t*)}.

For channel activation, deactivation, and inactivation, the linear part of the Kv1.1. ion channel model *G*(*s*) with three poles and two zeros is given by Equation (5), representing the TF in Laplace domain.

(5) $$G\left(s\right)=\frac{ℒ\left\{i_{i}\left(t\right)\right\}}{ℒ\left\{v_{i}\left(t\right)\right\}}=\frac{b_{2}s^{2}+b_{1}s+b_{0}}{a_{3}s^{3}+a_{2}s^{2}+a_{1}s+a_{0}}$$

Mathematical transformations can now be used to determine the differential equation of the system in the time domain, i.e., a third-order differential equation that describes the kinetic characteristics of the ion channel, representing the opening behavior of the channel at different voltage levels.

**Figure 4:**
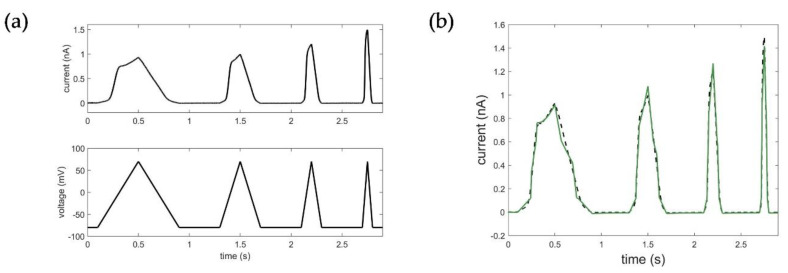
Results of the Kv1.1. STB model identification: (**a**) input voltage data using the ramp protocol in mV, output current data in nA representing the measured Kv1.1 macroscopic current; (**b**) optimization result of the STB model. Black: measured current data; green: simulated current data (RMSE_ramp_STB_ = 0.0364).

The transfer function TF in the time domain, thus, represents the so-called behavioral differential equation (BDE) and can be denoted as:

(6) $$a_{3}\overset{\ldots }{i\prime }\left(t\right)+a_{2}\ddot{i\prime }\left(t\right)+a_{1}\dot{i\prime }\left(t\right)+a_{0}i\prime \left(t\right)=b_{0}v\prime \left(t\right)+b_{1}\dot{v\prime }\left(t\right)+b_{2}\ddot{v\prime }\left(t\right)$$

where $a_{k},\;k\in \left(0,3\right)$ and $b_{k},\;k\in \left(0,2\right)$ are coefficients of TF and BDE, when all initial conditions are equal to zero. It is important to emphasize that all $a_{k}$ coefficients are positive, which can be explained by the transient response of the system, but also results from system identification. In terms of the former, we can conclude that the obtained ion channel model is “stable” according to the stability criteria in control theory. The model was obtained in MATLAB using the System Identification toolbox, [31] and estimated using PEM (prediction error minimization). Fitting results were determined by using RMSE values. Figure 4 shows the results of the Kv1.1 STB model parameterization.

If we look at the whole system, having the theoretical considerations in mind, the final Kv1.1 model is described by a nonlinear system with regularly coupled subsystems. Equation (7) represents the final model equation in continuous time description for simulation of the Kv1.1 current according to different voltage protocols.

$$v^{\prime }\left(t\right)=\{\begin{array}{c} 1.2\cdot v\left(t\right),\;\;\\ \begin{array}{c} 0.2\cdot v\left(t\right),\;\;\\ 0.1\cdot v\left(t\right),\;\; \end{array} \end{array}$$

(7) $$\begin{array}{l} \overset{\ldots }{i\prime }\left(t\right)+4321\;\ddot{i\prime }\left(t\right)+4.104\cdot 10^{8}\;\dot{i\prime }\left(t\right)+1.596\cdot 10^{9}\;i\prime \left(t\right)\\ \;\;\;\;\;=-6.268\cdot 10^{8}v\prime \left(t\right)+-1.754\cdot 10^{8}\;\dot{v\prime }\left(t\right)\\ \;\;\;\;\;+-1.269\cdot 10^{4}\;\ddot{v\prime }\left(t\right)\; \end{array}$$

$$i\left(t\right)=\;\{\begin{array}{c} -0.3\cdot i^{\prime }\left(t\right),\;\;\\ \begin{array}{c} -0.1\cdot i^{\prime }\left(t\right),\\ -0.01\cdot i^{\prime }\left(t\right), \end{array} \end{array}$$

One of the first and most important steps in system identification is the selection of the input based on prior knowledge and experiment design [27]. In comparison to voltage step protocols commonly used to determine the specific activation, deactivation, and inactivation properties of the ion channel, ramp protocols in turn provide a continuous recording of the overall dynamic behavior over a large voltage range, which is essential for reliable system identification. Based on the available experiments, the voltage data of the ramp protocol was, thus, used as the input function for system identification of the Kv1.1 STB model. Figure 4 shows the corresponding results of the estimation of the STB model. Detailed information on model parameterization can be found in Appendix A.

#### 2.4.2. The HMM-Based Kv1.1 Model

In order to represent the possible conformational states and structural changes underlying channel gating adequately and sufficiently, kinetic schemes of HMMs are derived based on the specific protein structure and known functional properties of the ion channel are additionally considered. The specific structure and investigated kinetic characteristics of the Kv1.1 channel that form the basis for model derivation are briefly summarized below.

**Figure 5:**
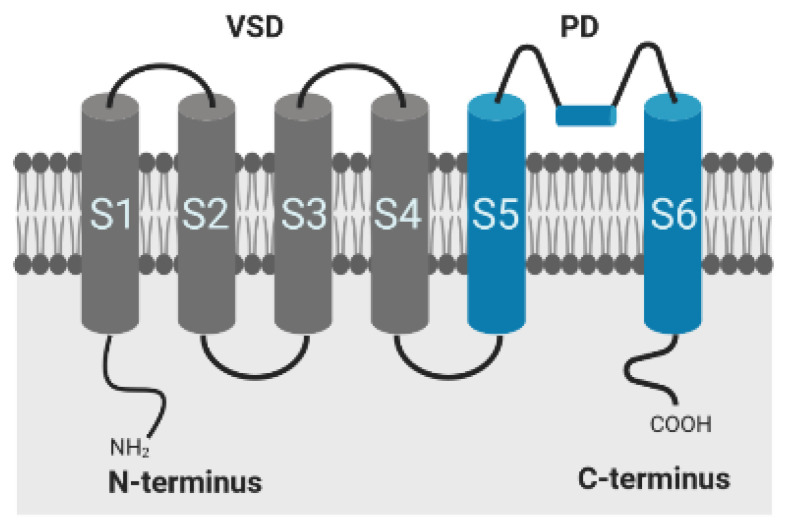
Alpha-subunit of the shaker-related voltage-gated potassium channel Kv1.1; VSD: voltage sensor domain; PD: pore domain. Created with BioRender.

The conductivity of voltage-gated potassium (Kv) channels depends on protein conformational changes in response to membrane depolarization [32]. The Kv pore-forming protein consists of 4 α-subunits, where each subunit is composed of six transmembrane segments (S1–S6) and intracellular N- and C-terminal domains, responsible for inactivation of the channel. The first four segments comprise the voltage sensor domain (VSD), segment S5, and S6 form the ion-conducting pore (PD) of the channel, as shown in Figure 5. Positively charged amino acids within the S4 segment trigger movements of the sensor in response to changes in membrane potential, which are transmitted to the pore via the S4–S5 linker for controlling the opening and closing of the channel [32,33,34]. Inactivation occurs by both a rapid N-type inactivation caused by the cytoplasmatic N-terminal sequence occluding the channel pore in the open state and by C-type inactivation, which is a slower time-dependent conformational change, leading to a narrowing of the outer mouth of the channel pore [34]. The α-subunits of Kv1.1 channel of mammalian cells lack the N-terminal sequence, but the proteins, however, exhibit a fast inactivation when complexed with subunits or auxiliary proteins that contain this domain and substitute the functionality, such as Kv1.4 or Kvβ1 and Kvβ3 [35,36,37,38]. In vivo, the channels are typically assembled with peripheral β-subunits, which modify the surface expression of these channels in addition to the gating behavior [36,37]. As recently demonstrated, physiological temperature equally provokes a fast inactivation in Kv1.1 channels, even in the absence of Kvβ1 and Kvβ3 subunits, emphasizing the important role of temperature on channel kinetics and function [15].

**Figure 6:**
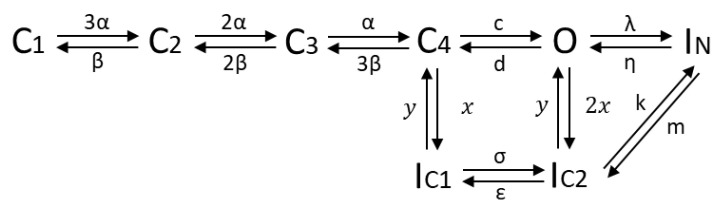
HMM of the Kv1.1 channel; C: closed; O: open; IC: slow inactivation; IN: fast inactivation.

Taking the knowledge of the protein structure and kinetic characteristics of the channel into consideration, a hidden Markov model with 8 states was defined (see Figure 6), consisting of 4 closed states (C), representing the 4 alpha subunits, all of which have to be in the open state before ions can pass; one open state (O); two inactivated states (IC) representing the slow inactivation that can occur from the closed and open state; one state accounting for the fast inactivation (IN), coupled to the open state. As found in several optimization runs, a better fit of the data was obtained when assuming a transition possibility between the fast and slow inactivation, suggesting a linkage of the two inactivation modes. Therefore, a direct transition path between IC2 and IN was considered in the final model approach [39].

Forward transition rates *α*, *λ*, *σ* and backward transitions *β*, *η*, *ε* are voltage-dependent and described by first-order exponential functions:

(8) $$\alpha \left(V\right)=\alpha_{1}.exp\left(\frac{V}{\alpha_{2}}\right)$$

(9) $$\beta \left(V\right)=\beta_{1}.exp\left(\frac{-V}{\beta_{2}}\right)$$

where *α_i_* and *β*_i_ represent specific gating parameters and *V* the applied voltage. *c*, *d*, *m*, *k*, *x*, and *y* denote rate constants without voltage-dependence. Defining $P_{S_{i}}\left(t\right)$ as the probability of being in a specific state *S_i_* at time *t* leads to the equation for the time evolution of the channels’ open probability $P_{O}\left(t\right)$ [2,40]:

(10) $$\frac{dP_{O}}{dt}=P_{C_{4}}\left(t\right)\cdot c+P_{I_{C2}}\left(t\right)\cdot y+P_{I_{N}}\left(t\right)\cdot \eta -P_{O}\left(t\right)\cdot \left(d+\lambda +2x\right)$$

where the first three terms represent transitions entering the open state O and the term furthest to the right transitions leaving the open state O.

Since HMMs model the current through a single ion channel, optimization based on measured whole-cell currents requires estimating the number of ion channels in addition to the model parameters for simulating the macroscopic current. For sufficiently large numbers of the same channel, the fluctuations in the stochastic opening of individual ion channels average out and the quantities in Equation (10) can be replaced by their macroscopic interpretation. Moreover, the probability of being in state $S_{i}$ can be interpreted as the fraction of channels in $S_{i}$. The transition probabilities become rate constants, $r_{i,j}$, which describe the number of channels that change from $S_{i}$ to $S_{j}$ in a given time period [2,40]. The macroscopic current $I_{Kv1.1}$ is given by the open probability *P_O_*, the ion channel number *N_c_*, the single channel conductance *g*_Kv__1.1_, and the reversal potential *E_K_*:

(11) $$I_{\mathrm{Kv}1.1}=g_{\mathrm{Kv}1.1}.N_{c}.P_{O}.\left(V-E_{K}\right)$$

The rate constants were parameterized using a particle swarm optimization (PSO) algorithm from the Global Optimization Toolbox (MathWorks Inc., Natick, MA, USA) based on averaged activation and deactivation measurements. The number of sample cells considered for the activation and deactivation currents differed, and since the magnitude of the macroscopic current varied considerably from cell to cell, the magnitude of the macroscopic currents at similar voltage levels also showed considerable deviations between patch clamp experiments assessing the activation and deactivation characteristics. To account for these variations, the number of ion channels *N_c_* was individually optimized for each measurement protocol [41]. For the given dataset, the channel number was determined as N_c_act_ = 3088 for the measured activation and N_c_deact_ = 2588 for the deactivation currents. The final model parameters are summarized in Table 1.

**Figure 7:**
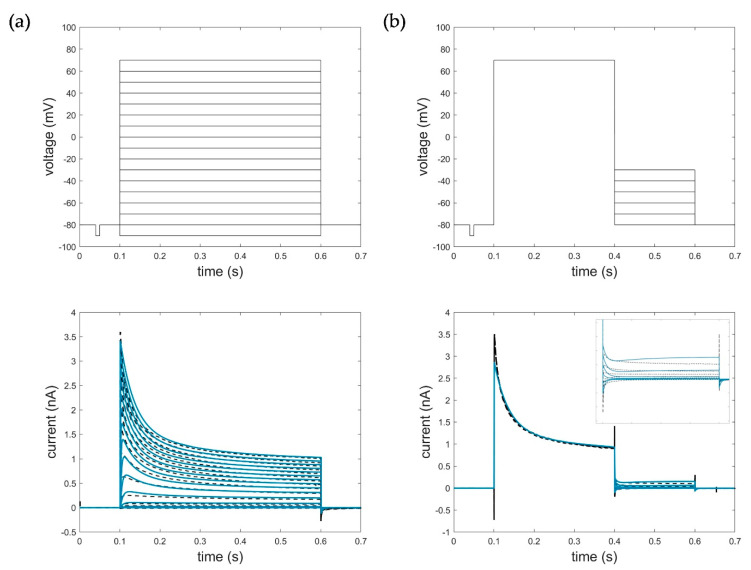
Optimization result of the HMM for (**a**) activation data (−90 mV to 70 mV, ∆V = 10 mV, RMSE_act_HMM_ = 0.0714) and (**b**) deactivation data with zoomed deactivation pulses (−80 mV to −30 mV, ∆V = 10 mV, RMSE_deact_HMM_ = 0.1098). Black: measured current data; blue: simulated current data. The first line represents the respective voltage step protocols.

Figure 7 shows the corresponding simulation results of model parametrization (RMSE_act_HMM_ = 0.0714, RMSE_deact_HMM_ = 0.1098). For detailed information on model parametrization and simulations, see Appendix A.

The basic idea of HMMs is to model the specific changes in the conformational states of the protein represented by the different states in the model. To determine whether the transitions and the occupancy of states in the HMM in response to a stimulus corresponded to the underlying kinetics of the channel, we simulated the model stochastically by generating a random sequence of states using the *hmmgenerate* function in MATLAB (MathWorks Inc.).

**Figure 8:**
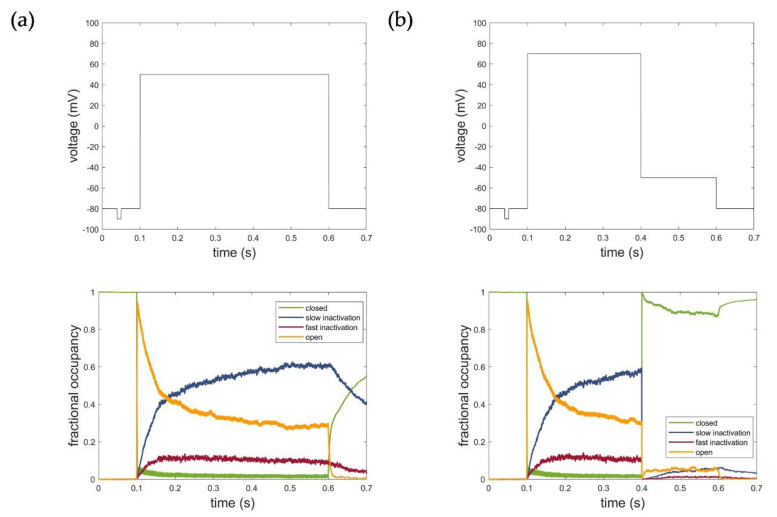
Fractional occupancy plot of hidden Markov model simulations for (**a**) activation protocol at voltage level of 50 mV and (**b**) deactivation protocol at voltage level of −50 mV. C: closed state; O: open state; IC: slow inactivation states; IN: fast inactivation states. The first line represents the respective voltage step protocols.

Figure 8 illustrates the corresponding fractional occupancy plots based on the simulation of 2000 individual Kv1.1 channels for both voltage protocols used for parametrization. Consistent with the kinetics, the simulations revealed that the inactivation occurred through transitions from the open state to states representing the fast and slow inactivation. Deactivation, in turn, occurred mainly through transitions from the open and inactivated states to the closed states in the model. The fractional occupancy plots, thus, impressively demonstrated the reliable modeling of the actual channel kinetics and confirmed and verified the developed Kv1.1 hidden Markov model.

#### 2.4.3. The HH-Based Kv1.1 Model

To model the specific Kv1.1 channel conductance, Ranjan et al. [15] adapted the original model of Hodgkin and Huxley [42] (see Appendix D) describing the non-linear potassium conductance in the squid giant axon and added an additional gate h to account for channel inactivation, with single gates for activation and inactivation (see Equation (12)).

(12) $$I_{\mathrm{Kv}1.1}=\bar{g_{\mathrm{Kv}1.1}}m^{p}h^{q}\left(V-E_{K}\right)\;p=q=1\;$$

with

$$\frac{dm}{dt}=\frac{m_{\infty }-m}{\tau_{m}}$$

$$\frac{dh}{dt}=\frac{h_{\infty }-h}{\tau_{h}}$$

The process of model adaption and fitting of the Kv1.1 HH model can be briefly summarized as follows: the steady state variables $m_{\infty }$ and $h_{\infty }$ were fitted to single Boltzmann functions:

(13) $$m_{\infty }=\frac{1}{1+e^{\frac{V-V_{1/2}}{-k}}}$$

(14) $$h_{\infty }=\left(1-A\right)+\frac{A}{1+e^{\frac{V-V_{1/2}}{k}}}$$

where *V*_1/2_ denotes the half activation and inactivation voltage, *k* the slope factor, and *A* the starting point. The time constant for activation $\tau_{m}$ was fitted by two Boltzmann equations, and a single Boltzmann equation was again used for $\tau_{h}$:

(15) $$\tau_{m}=\left(A1+\frac{B-A1}{1+e^{\frac{V-c}{d}}}\right)+\left(A2+\frac{B-A2}{1+e^{\frac{V-c}{d}}}\right)$$

(16) $$\tau_{h}=\left(A+\frac{B}{1+e^{\frac{V-c}{d}}}\right)$$

Normalized conductivities of measured current traces from activation voltage step protocols between −40 mV and 50 mV were fitted for each cell and temperature level (15 °C, 25 °C, and 35 °C). Single-cell models that had a residual sum of squares (RSS) less than 0.36 were considered, and the median values for each gating parameter and temperature level were used for the final model [15].

To account for the temperature-dependent conductivity of the Kv1.1 channel, the median gating parameters of $h_{\infty }$, $\tau_{m}$ and $\tau_{h}$ obtained at each temperature were further fitted with Q10 functions. In comparison, $m_{\infty }$ was considered to be temperature-independent, despite different values of gating parameters in the revised HH model. The model equations and gating values of the proposed model by Ranjan et al. [15] are given in Equations (17)–(23).

(17) $$m_{\infty }=\frac{1}{1+e^{\frac{V-\left(-14.16\right)}{-10.15}}}$$

(18) $$h_{\infty }=\left(1-h_{\infty }Q_{10}\right)+\frac{h_{\infty }Q_{10}}{1+e^{\frac{V-\left(-31.0\right)}{5.256}}}\;\mathrm{with}\;h_{\infty }Q_{10}=\left(0.032\cdot {}^{\circ}C\right)-0.365$$

(19) $$\tau_{m}=\frac{m_{\tau \_Func\left(V\right)}}{m\tau Q_{10}}\;\mathrm{with}\;m\tau Q_{10}={\left(7.54\cdot e^{\frac{-V}{379.7}}\cdot e^{\frac{-{}^{\circ}C}{35.66}}\right)}^{\frac{{}^{\circ}C-25}{10.0}}\;$$

(20) $$\tau_{h}=\frac{86.86+\frac{408.78}{1+e^{\frac{V-\left(-13.6\right)}{7.46}}}}{h\tau Q_{10}}\;\mathrm{with}\;h\tau Q_{10}=2.7^{\frac{{}^{\circ}C-25}{10.0}}$$

(21) $$m_{\tau \_Func\left(V\right)}=sig1+sig2\;sigswitch=\frac{1}{1+e^{\frac{V-\left(-46.7\right)}{3}}}$$

(22) $$sig1=sigswitch\frac{amp1}{1+e^{\frac{V-V1_{1/2}}{-slope1}}}$$

$$amp1=52.7,\;V1_{1/2}=-49.87,\;slope1=5.0$$

(23) $$sig2=\left(1-sigswitch\right)\cdot offset+\frac{amp2-offset}{1+e^{\frac{V-V2_{1/2}}{slope2}}}$$

$$amp2=15.98,\;V2_{1/2}=-41.64,\;slope2=24.99,\;offset=0.9$$

Table 2 summarizes the key features of the HH, HMM, and STB models, including the number of unknown parameters to be optimized, the extent of the mathematical description of the models, and the data used for model parameterization. Detailed information on model parameterization is provided in Appendix A.

### 2.5. Evaluation, Verification, and Comparison of the Three Model Approaches

**Figure 9:**
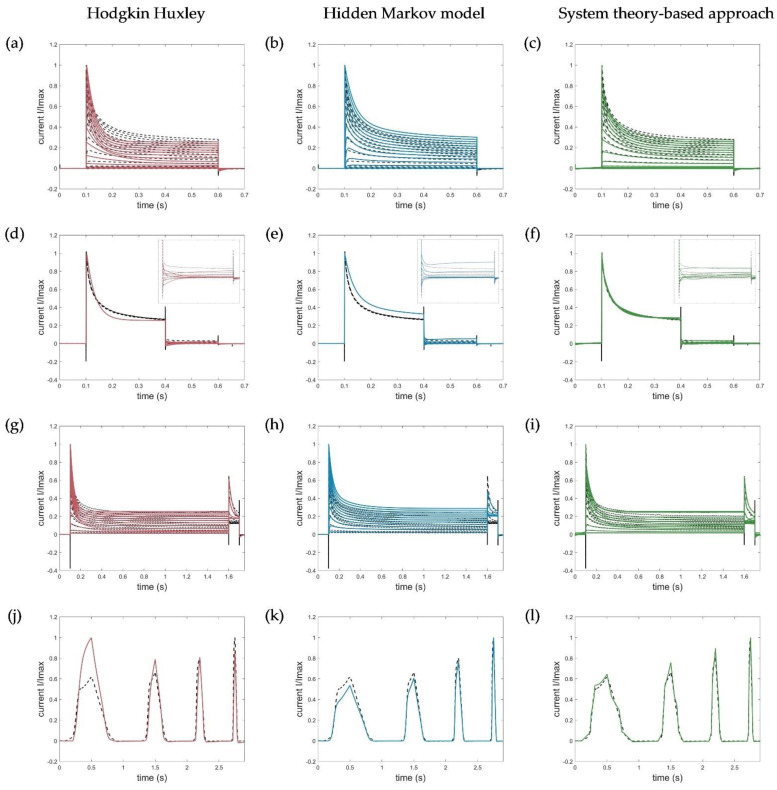
Model simulations of (**a**–**c**) activation protocols (−90 to 70 mV, ∆V = 10 mV), (**d**–**f**) deactivation protocols with zoomed deactivation pulses (−80 to −30 mV, ∆V = 10 mV), (**g**–**i**) inactivation protocols (−80 to −70 mV, ∆V = 10 mV), and (**j**–**l**) ramp protocols by the HH, HMM, and system theory-based approaches, respectively. Ion channel numbers N_c_ used for simulation of the macroscopic current with the HMM model are (**b**) N_c_act_ = 3088, (**e**) N_c_deact_ = 2588, (**h**) N_c_inact_ = 2588, and (**k**) N_c_ramp_ = 2388. Voltage step and ramp protocols for simulations are shown in Figure 1.

For the model evaluation and verification, different voltage protocols performed to determine the channel kinetics (see Figure 1) were simulated using each of the three model approaches and compared accordingly. Corresponding simulation results of the Hodgkin–Huxley formalism, the developed hidden Markov model, and the system theory-based approach for the activation, deactivation, inactivation, and ramp protocols are shown in Figure 9.

Since, in contrast to the developed approaches, the HH model was parametrized to fit the normalized currents and, thus, defined only for them, the simulated currents of the activation, deactivation, and inactivation protocols were each normalized to the maximum measured current at 70 mV for comparison. For the ramp currents, the maximum value of the entire trace was used for normalization.

The goodness of fit of the simulated current curves was evaluated directly using the root mean square error (RMSE) and averaged over all voltage levels for both the normalized (RMSE_norm_) and absolute (RMSE_abs_) currents (Equation (24)).

(24) $$\mathrm{RMSE}=\sqrt{\sum {\left(I_{\mathrm{Kv}1.1\_\mathrm{model}}\left(t\right)-I_{\mathrm{measured}}\left(t\right)\right)}^{2}/N}$$

The developed hidden Markov model and the system theory-based model outperformed the HH model in terms of data fitting and reproduced the specific activation, deactivation, and especially the recorded ramp currents very accurately. Remarkably, the activation currents simulated with the STB model were almost identical to the measured current, as shown by the obtained RMSE value, summarized in Table 3.

The deviations of the HMM in the obtained deactivation curve at −30 mV, showing an increase in the current after the corresponding deactivation, could be explained by the high-voltage level, which naturally led to an activation of the channels (see Figure 9e). In turn, the disturbances in the STB model resulted from the capacitive spikes that were not filtered out and removed from the measured current traces. Because of these spikes, the model did not reproduce the raw output data more accurately (Figure 9f).

**Figure A1:**
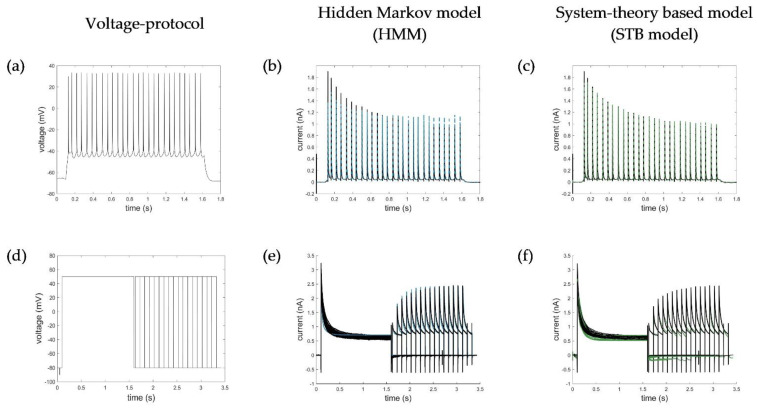
Simulation of additional (**a**) action potential (AP) and (**d**) recovery protocols with the HMM (**b**,**e**) and STB models (**c**,**f**). Corresponding RMSE values for simulation of the AP protocol were RMSE_AP_HMM_ = 0.0435 and RMSE_AP_STB_ = 0.0321; for the recovery protocol: RMSE_recovery_HMM_ = 0.1200 and RMSE_recovery_STB_ = 0.0831. RMSE values excluding the depolarization pulses of recovery protocols calculated from 1.5 s to the end of the test pulses were RMSE_recovery_HMM_ = 0.0777 and RMSE_recovery_STB_ = 0.0945. Ion channel numbers N_c_ used for simulation of the macroscopic current with the HMM model were (**b**) N_c_AP_ = 2900 and (**e**) N_c_recovery_ = 2600.

Similarly, the fast inactivation could be modelled with high precision by the newly developed STB compared to the HH approach, that revealed a too strong and prolonged inactivation. A slightly higher RMSE, in turn, was obtained for the simulation of the inactivation curves by the HMM due to moderate deviations of the absolute currents. In general, however, the kinetics correlated well with the measured current dynamics, which was also an acceptable modeling result for the developed HMM. Thus, both models, which were parametrized on a few single-current curves only (see Table 2), were suitable for different input functions and were able to simulate the specific Kv1.1 current, which serves as the first step in verifying and proving the validity of the model. Additional simulations using the HMM and STB approaches performed with action potential (AP) and recovery protocols can be found in Appendix B, Figure A1, which also shows useful simulation results and confirms the potential of the new STB-based modeling approach.

For a thorough evaluation of the accuracy of the models, basic electrophysiological parameters describing the activation, deactivation, and inactivation properties were extracted and compared.

Activation characteristics were evaluated using the conductance voltage relation and the time constant for activation, measured by the activation protocols performed. For this purpose, the normalized conductivities calculated from the peak currents at each voltage step (Equation (25)) were plotted as a function of the test pulse voltages and fitted to a Boltzmann function (Equation (26)):

(25) $$G=\frac{I_{peak}}{\left(V-E_{K}\right)}$$

(26) $$\frac{G}{G_{max}}=\frac{1}{exp^{\frac{V-V_{1/2\_act}}{kact}}}$$

where *G_max_* is the maximal conductance measured at a voltage step of 70 mV, *V*_1/2_*act*_ the hemi-activation voltage, and *k_act_* the slope factor. The activation time constant was determined by fitting a single exponential function to each individual current curve from the start of the stimulus to the peak current:

(27) $$y=1-exp^{\frac{-t}{\tau_{act}}}$$

Tail currents evoked by hyperpolarization pulses following a depolarization step of 300 ms duration were measured and analyzed to determine the deactivation properties. Each individual tail current obtained by the deactivation protocol was fitted to a single exponential function to estimate the time constant of deactivation:

(28) $$y=A_{1}\cdot exp^{\frac{-t}{\tau_{deact/inact}}}+A_{2}$$

with τ as the time constant of deactivation and $A_{1}$ and $A_{2}$ the initial and final values, respectively.

Inactivation characteristics were determined based on the steady-state availability protocols performed that included conditioning pulses of longer duration at different voltage steps to establish a steady-state inactivation after channel activation, followed by a depolarizing voltage step (to activate channels still in an activatable state). The inactivation time constants were calculated based on the activation pulse by fitting a single exponential function from peak to steady state for each current trace according to Equation (28). The half-inactivation voltage *V*_1/2_*inact*_ and the slope factor of inactivation *k_inact_* were, again, calculated by fitting the normalized peak currents of the depolarizing voltage step to a Boltzmann function according to Equation (29):

(29) $$\frac{I}{I_{max}}=A_{1}+\frac{A_{2}-A_{1}}{1+exp^{\frac{V-V_{1/2\_inact}}{k_{inact}}}}$$

Slow voltage ramps were used to determine the voltage at which the channels had maximum conductance *V*_max_cond_. Following Ranjan et al. [15], the peak value during the rising phase of the first ramp was used as the parameter *V*_max_cond_.

**Figure 10:**
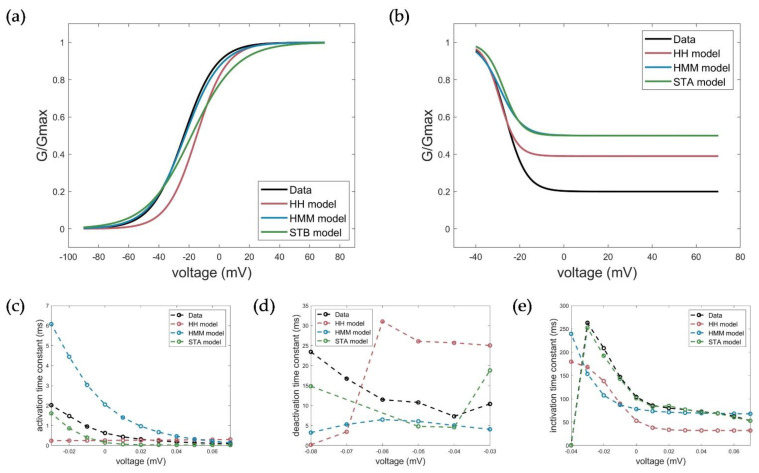
Measured (black) and simulated electrophysiological parameters by the HH (red), HMM (blue), and STB (green) models. (**a**) Conductance plot of activation, (**b**) conductance plot of inactivation, (**c**) time constant of activation, (**d**) time constant of deactivation, and (**e**) time constant of inactivation.

The calculated and extracted electrophysiological parameters are shown in Figure 10 and summarized in Table 3.

The measured activation characteristics with a half activation voltage of *V*_1/2_act_measured_ = −22.45 mV and slope factor *k*_act_measured_ = 10.81 mV were best reproduced by simulations with the HMM (*V*_1/2_act_HMM_ = −22.64, *k*_act_HMM_ = 11.82 mV). For the STB model, the curve and, thus, the half activation voltage were slightly shifted towards a more depolarized value, but comparable results to the HH model could be obtained with *V*_1/2_act_STB_ = −18.39 and *k*_act_STB_ = 14.97 mV relative to *V*_1/2_Act_HH_ = −14.94 and *k*_act_HH_ = 9.913 mV. With respect to the activation time constant, both the HMM and the STB model better reflected the actual voltage-dependent dynamics of activation by showing a faster activation time at higher clamp voltages and a slower activation as the voltage decreased, compared to the HH model with the same time constant over the entire voltage range. However, the activation in the STB model was instantaneous and, thus, somewhat too fast, while the activation in the HMM, especially at lower voltages, was too slow compared to the measured values. The simulation results for the deactivation of the HMM and STB models revealed a slower deactivation, but they, again, better reflected the measured deactivation behavior compared to the HH model, as shown by the determined deactivation time constants (see Figure 10 and Table 3).

The model simulations of the inactivation curves showed a slightly better, but comparable half inactivation voltage compared to the HH model with respect to the measured parameters for the HMM model (Figure 10b). In contrast, the STB approach again outperformed the accuracy of the HMM and HH models, and showed a nearly perfect fit of the measured inactivation time constants; see Figure 10e and Table 3.

Taking all the results obtained into consideration, both the newly developed HMM and the STB approach provided an accurate modeling of the channel kinetics that better reflected the underlying dynamics of the channel in response to various input functions than the established HH model used here as benchmark or state-of-the-art model. In particular, the HMM and the STB models provided two valuable new approaches for ion channel modeling and the simulation of the Kv1.1 current at a physiological temperature.

## 3. Discussion

Single-channel modeling is a central component of computational electrophysiology. Today, extensive experimental investigations and a steadily growing body of knowledge about ion channels enable the development of highly detailed models that simulate the specific gating behavior and the bioelectric properties of ion channels. The increasing biophysical detail, however, also inevitably leads to high computational costs, which, to some extent, limit both the construction and the application of complex whole-cell models, especially for simulations on the tissue and organ level. Hence, while detailed HMMs that map the protein structure and better address the processes behind channel gating are mainly considered in biomolecular and pharmacological research, HH models, for example, are still the golden standard in neuroscience, since they provide a low computational cost method and, thus, a high integrability into complex models to represent the electrophysiological activities of cells, tissue layers, and up to whole organs.

Beyond conventional methods, following the phenomenological approach of Hodgkin and Huxley, we proposed for the first time a new system theory-based concept of deterministic ion channel modeling and the simulation of ion currents, which provide an easy-to-use method with remarkable performance and accuracy, especially with respect to the structurally comparable HH models. Using the example of Kv1.1 (KCNA1) delayed rectifier channels, which are strongly expressed in the central and peripheral nervous system and “regulate” neuronal subthreshold excitability and spike initiation [20,21,22,24], the newly introduced method was compared with the concepts of the HH model and HHM, and evaluated on several parameters relevant in the computational modeling of cellular electrophysiology.

### 3.1. Model Accuracy

**Figure A2:**
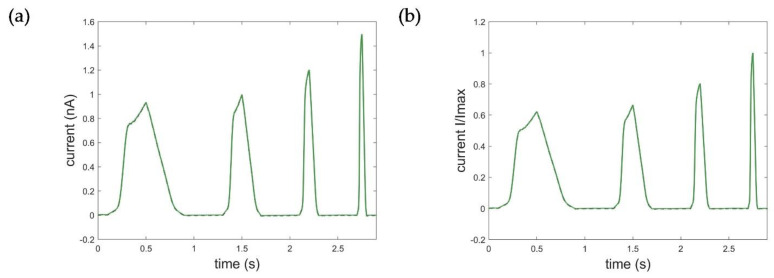
Optimization result of the STB model using 10 breakpoints for stepwise linearization of the non-linear input and output function. (**a**) Absolute ramp currents (RMSE_ramp_STB_abs_ = 0.0023) and (**b**) normalized ramp currents (RMSE_ramp_STB_norm_ = 0.0013). Black: measured current data; green: simulated current data.

The introduced STB model, parametrized on the ramp data only, allows the accurate simulations of the specific kinetics of the Kv1.1 channel and fits almost perfectly with the measured currents for the different voltage protocols performed (see Figure 9 and also Figure A1), even in a currently highly simplified and well-interpretable form where only two breakpoints were used to approximate the nonlinear input and output function. The accuracy could be further improved by considering additional breakpoints. Figure A2 in Appendix C shows an example simulation of the ramp data using 10 breakpoints in the STB model with an almost perfect fit. However, a higher number of breakpoints resulted in a more complex system description, represented by an even higher order and a less interpretable differential equation in the time domain.

As shown by a direct comparison with a recently published HH model of the Kv1.1 and the new HMM model developed here based on the same experimental data, the STB model outperformed the established models in accuracy and better reproduced the specific activation, deactivation, and inactivation properties of Kv1.1 channels at a physiological temperature. It is important to note that the HH model, used as a benchmark for comparison and model validation, also accounted for the temperature-dependent modulation of the channel kinetics and was parameterized based on the activation curves of different temperature levels, i.e., 15 °C, 25 °C, and 35 °C. For this reason, the HH model represented an average best model for simulating the Kv1.1 current within this temperature range, but did not perfectly match the measured currents at a single temperature. However, simulation results that were within the deviations of the HH model were considered sufficiently reasonable and valid.

Comparable results were obtained for the newly developed HMM in terms of fitting the experimental data to the HH model. The optimization of the HMM to the activation data only allowed an almost perfect simulation of the activation curves, while the deactivation and inactivation characteristics were not represented at all. Furthermore, parametrization based on the ramp curves, as performed for the STB approach, did not lead to a satisfying modeling result. The HMM model was, thus, finally parametrized based on the activation and deactivation curves, which also allowed the inactivation to be adequately represented by the model approach and acceptable model simulations of all voltage-protocols (see Figure 9 and Figure A1). However, the model showed a lower accuracy with regard to the inactivation characteristics. Therefore, in a next step, more attention should be paid to the inactivation path, e.g., by considering additional inactivation protocols in the model parameterization, experimental investigations, and an appropriate redefinition of the number of states, representing the slow and fast inactivation, in order to improve the validity of this newly introduced hidden Markov-based Kv1.1 model.

### 3.2. Model Complexity, Explainability, and Adaptability

Compared to the HMM, but similar to Hodgkin and Huxley, the STB approach is entirely data-driven and does not take into account any electrophysiological knowledge, which, currently, does not allow for inference or insights into the inherent channel gating mechanisms by the model approach. By contrast, even at a highly simplified level, the kinetic schemes of HMM, which map the transitions between different conformational states, offer better explainability compared to the HH and STB models, and the study of specific modifications in the opening and closing behavior of channels, as particularly needed, for example, in pharmacological studies. Moreover, since HMMs describe the dynamics of single channels, they provide a high degree of flexibility and allow its application to different datasets with varying dynamics or current amplitudes by adjusting the rate constants or number of ion channels. HH models, as well as the newly introduced STB approach, always represent the measured macroscopic currents and are valid only for a specific dataset. Therefore, a direct adoption to other experimental data, sample populations, or cells with varying ion channel compositions, is usually not possible without an appropriate and comprehensive reparameterization.

However, the proposed HMM represents a simplified kinetic scheme derived solely on the basis of macroscopic currents and does not take into account further electrophysiological studies such as single-channel recordings or structural studies of protein conformation, which limits the degree of the explainability and adjustability of this first HMM of the Kv1.1 channel. Furthermore, with respect to the inactivation characteristics, no characterization of the slow and fast inactivation was performed, e.g., an assessment of the respective proportion using specific blockers. Additionally, the assumption of a possible transition between and, thus, an interaction of the slow and fast inactivation implemented by a cross-link between the IC2 and IN1 states was based only on achieving a better modeling result as shown in several optimization runs, but without experimental validation. Thus, the states in the model do not correspond to the actual protein conformational states and microscopic conformational changes of the protein, but can be viewed as aggregates of molecular configurations grouped into a set of distinct functional states separated by large energy barriers [1].

Despite the aforementioned simplifications, the HMM model allowed an accurate and reliable simulation of the different measured kinetics, as shown by the occupancy diagrams. The occupancy of states was consistent with the measured and known kinetics, which confirmed the validity of the proposed kinetic scheme and parameterization for modeling the kinetics of the Kv1.1 channel.

### 3.3. Computational Burden

Together with the complexity and level of detail, the high computational cost is one of the major drawbacks limiting the application of HMM. Even simplified kinetic schemes, such as the one developed in this work, include a great number of parameters and states that are implemented in the model by a set of first-order differential equations, implying the need for a very a high computational effort not only in terms of simulation runtime, but also for parametrization. In contrast to the HMM and HH methods, the system theory-based approach significantly reduced the typically huge set of differential equations in the HMM approach to one single higher-order differential equation that describes the current-voltage relation of the ion channels as a nonlinear system with regularly coupled subsystems. This enormously reduces the computational cost for parameterization and model simulation. Together with the remarkable model accuracy, this represents the main advantage of the newly developed STB model compared to the traditional modeling approaches in electrophysiology.

For HMM in particular, the large number of parameters relative to the comparatively few data also increases the risk of overfitting and, thus, limits the predictive power and reliable simulation of additional data. Therefore, it makes sense to keep the HMM as simple as possible by involving different measurement protocols in model optimization. However, if more data were included in the model optimization, the time for parameterization would increase again. For the developed HMM parametrized on the activation and deactivation curves, each optimization run took about 30 h on a high- performance computer with 12 cores working in parallel for model parametrization. By contrast, the parameterization of the STB model function based solely on the ramp curves was, for example, performed in less than 10 min using the same computer infrastructure with MATLAB (System Identification toolbox, MathWorks Inc.).

Compared to the HH models, HMM also had, on average, a higher computation time, even with a smaller number of states, as shown, for example, in a study by Andreozzi et al. [1], which yielded a 5% higher runtime of a simplified HMM compared to the corresponding HH model. However, given the simulation results obtained, which showed excellent accuracy compared to the HH approach, the increased computation time was considered to be acceptable. For our Kv1.1 simulations, the runtime of an example cell with 3500 individual Kv1.1 channels was about 20 times higher for the HMM than for the HH and the STB model, with the latter requiring less than 1 s.

### 3.4. Experimental Data for Model Parameterization

It is important to note that electrophysiological studies are generally time-consuming, and obtaining representative, quality-assured results usually requires a high experimental effort. The experimental data used in this study are publicly available and include measured whole-cell currents from transfected cells, stably expressing Kv1.1 channels recorded with different voltage protocols. For phenomenological modeling, the data required for model parametrization were rather small and comparable for all modeling approaches examined in this work. They included measured macroscopic currents from patch clamp recordings with standard voltage step protocols to characterize the activation, deactivation, and inactivation characteristics. In order to fully characterize the kinetic properties and improve the validity of HMMs, however, extensive experimental investigations are required, such as single-channel patch clamp measurements, determination of fast and slow inactivation and possible cross links, or structural studies to gain a deeper understanding about the protein conformational states. All these together increase the experimental effort required for HMM development and validation in general enormously compared to the HH model or, in particular, to the newly proposed system theory-based modeling approach.

### 3.5. Which Method Should Now Be Chosen? When, How, and Why?

The three different modeling approaches presented in this work all have both strong advantages and disadvantages, and should always be selected with respect to the particular application. Table 4 summarizes the three modeling approaches by qualitatively comparing the key parameters in computational electrophysiology.

The system theory has been an established tool for modeling physical or biological processes for decades, and it is used traditionally in the field of control engineering. In this work, we introduced the concept of a transfer function for the kinetic characterization of single ion channels for the first time. We investigated the extent to which its properties could be used to simulate the activation, deactivation, and inactivation of channels without knowing the intrinsic biological and physical mechanisms, but only using the data characteristics of the input and output function of the “system”, which is presented by only the one third-order differential equation, taking the input and output nonlinearities into account. Today, available software tools, such as MATLAB, allow for an easy and automated characterization of the transfer function of the biological system, enabling simple and fast model parameterization compared to the conventional methods such as the HH model and HMM. With this easy to use parameterization strategy, this strongly data-driven modeling approach can be adapted simply to different datasets of sample populations with varying ion channel composition, and could make the system theory-based modeling approach the method of choice for high-performance simulations at the tissue and organ level. Further investigations could show whether and to what extent this concept can also be applied to other ion channel types with divergent kinetics, such as channels with a slow inactivation (e.g., Kv3.1) or constant activation (e.g., Kv7.1), after an appropriate system identification.

In contrast, by embedding knowledge from biophysical and structural studies, the HMM allows a detailed modeling of the specific functionality and structural changes underlying channel gating, representing possible dependencies of activation and inactivation, transitions from closed to inactivated states, or multistep activation processes. In particular, ligand- or second messenger-dependent changes as well as drug-induced effects on specific conformational states and, thus, on the functionality and kinetics of the channel can be investigated at the microscopic and macroscopic level by appropriate kinetic schemes, as ultimately required in pharmacological or molecular–biological investigations. For these applications, the Markov models, which take into account the inherent gating properties and better address the stochastic gating behavior, represent a perfectly suitable method despite the higher experimental effort and computational load [1,11]. Moreover, the HMM with a sufficient complexity and low computational cost used in whole-cell applications can overcome the limitations of the currently most widely used HH approaches, for example, by better accounting for the complex interplay of ion channels, calcium dynamics, or specific responses to changing environmental conditions such as the temperature, pH, or ionic composition. To this end, HMMs are increasingly considered for detailed modeling approaches to further improve the reliability and validity of complex single-cell applications. However, we can expect that if extensive experimental data-representing mechanisms such as drug-induced effects, changes to environmental conditions, or intracellular ionic compositions are available, appropriate STB models, because of their simple parameterization, could also be introduced.

In summary, the system theory-based modeling approach combines the positive features and properties of both the HH model and HMM. The proposed concept outperformed the HH model and HMM in accuracy, although it strongly abstracted the underlying electrophysiological mechanisms, while overcoming the current computational limitations of the HMM. In particular, for applications requiring high computational power, this newly introduced modeling approach offers a promising new possibility that could be used alongside or even instead of HH-based ion channel models in computational electrophysiology, while further improving the simulation accuracy and runtime. Thus, beyond single-cell applications, STB models have high potential to further improve the simulation performance of complex cell and organ models and may represent a valuable tool in the field of next-generation in silico electrophysiology.

## Figures and Tables

**Table 1:** Parameters of the Kv1.1 hidden Markov model.

Rate Constants and Parameters					
*α*_1_	951.2464 s^−1^	*λ*_1_	14.1140 s^−1^	*σ*_1_	3.8031 s^−1^
*α*_2_	0.03 V	*λ*_2_	20.2499 V	*σ*_2_	11.8850 V
*β*_1_	395.7896 s^−1^	*η*_1_	49.9528 s^−1^	*ε*_1_	58.364 s^−1^
*β*_2_	0.0501 V	*η*_2_	5 V	*ε*_2_	55.3568 V
*c*	799,720 s^−1^	*k*	370.9594 s^−1^	*x*	1.6056 s^−1^
*d*	38,916 s^−1^	*m*	1199.6 s^−1^	*y*	0.0822 s^−1^
*E_K_*	−0.065 V	*g*_Kv__1.1_	8.7 pS		
N_c_act_	3088	N_c_deact_	2588		

**Table 2:** Model characteristics and data involved in model parameterization.

	*HH*	*HMM*	*STB*
**unknown parameters**	22	20	7
**mathematical description of the model**	2 first-order differential equations	8 first-order differential equations	1 third-order differential equations
**parameterization data**	activation single-cell measurements	activation and deactivation average	ramp average
**number of cells**	56	60 (activation) 37 (deactivation)	54
**voltage range**	−40 to +50 mV	−90 to +70 mV (activation) −80 to −30 mV (deactivation)	−80 to +70 mV
**total sweep number** **considered**	10	13: −50 to +70 mV (activation) 6: −80 to −30 mV (deactivation)	1
**time for parameterization/system identification**	data not available	30 h	5–10 min

**Table 3:** Electrophysiological parameters.

Temp 35 °C		Experimental Data	Simulated Data		
			HH	HMM	STA
**activation**					
***V*_1/2_*act*_**	(mV)	−22.45	−14.94	−22.64	−18.39
***k_act_***	(mV)	10.81	9.913	11.82	14.97
** $\tau_{act\_mean}$ **	(ms)	0.5493	0.5766	2.2449	0.2706
** $\tau_{act\_70mV}$ **	(ms)	0.09283	0.3036	0.1391	0.01875
** $\tau_{act\_60mV}$ **	(ms)	0.1135	0.2949	0.2125	0.02084
** $\tau_{act\_50mV}$ **	(ms)	0.1403	0.2865	0.3157	0.02339
** $\tau_{act\_40mV}$ **	(ms)	0.1791	0.2783	0.4613	0.02634
** $\tau_{act\_30mV}$ **	(ms)	0.2351	0.2703	0.668	0.02994
** $\tau_{act\_20mV}$ **	(ms)	0.3148	0.2629	0.9654	0.03359
** $\tau_{act\_10mV}$ **	(ms)	0.4343	0.2567	1.401	0.06175
** $\tau_{act\_0mV}$ **	(ms)	0.6244	0.2524	2.052	0.1491
** $\tau_{act\_-10mV}$ **	(ms)	0.9504	0.2486	3.033	0.3984
** $\tau_{act\_-20mV}$ **	(ms)	1.476	0.2430	4.437	0.8607
** $\tau_{act\_-30mV}$ **	(ms)	2.02	0.2374	6.077	1.613
**RMSE_norm_** **RMSE_abs_**			0.0326 -	0.0213 0.0714 *	0.0138 0.0381
**deactivation**					
** $\tau_{deact\_mean}$ **	(ms)	13.3627	18.5689	5.0230	10.76
** $\tau_{deact\_-30mV}$ **	(ms)	23.42	0.1704	3.236	14.86
** $\tau_{deact\_-40mV}$ **	(ms)	16.75	3.433	5.282	-
** $\tau_{deact\_-50mV}$ **	(ms)	11.49	31.03	6.491	-
** $\tau_{deact\_-60mV}$ **	(ms)	10.79	26.07	6.058	4.793
** $\tau_{deact\_-70mV}$ **	(ms)	7.306	25.68	5.019	4.564
** $\tau_{deact\_-80mV}$ **	(ms)	10.42	25.03	4.052	18.82
**RMSE_norm_** **RMSE_abs_**			0.0429 -	0.0627 0.1098 *	0.0283 0.0985
**inactivation**					
***V*_1/2_*inact*_**	(mV)	−26.46	−29.12	−28.95	−27.37
***k_inact_***	(mV)	4.755	3.882	5.04	4.074
** $\tau_{inact\_mean}$ **	(ms)	102.1077	71.9092	96.4150	99.1621
** $\tau_{inact\_70mV}$ **	(ms)	53.22	32.14	68.15	53.2
** $\tau_{inact\_60mV}$ **	(ms)	63.13	32.14	68.45	60.65
** $\tau_{inact\_50mV}$ **	(ms)	69.17	32.17	68.85	68.74
** $\tau_{inact\_40mV}$ **	(ms)	72.65	32.25	69.41	71.38
** $\tau_{inact\_30mV}$ **	(ms)	77.25	32.57	70.25	77.26
** $\tau_{inact\_20mV}$ **	(ms)	80.26	33.79	71.6	85.05
** $\tau_{inact\_10mV}$ **	(ms)	85.9	38.27	73.89	83.26
** $\tau_{inact\_0mV}$ **	(ms)	104.3	53.18	78.19	101.5
** $\tau_{inact\_-10mV}$ **	(ms)	147.1	89.9	87.19	143.1
** $\tau_{inact\_-20mV}$ **	(ms)	208.7	138.4	107.7	192.4
** $\tau_{inact\_-30mV}$ **	(ms)	263.1	168.4	153.8	252.6
** $\tau_{inact\_-40mV}$ **	(ms)	0.5125	179.7	239.5	0.8051
**RMSE_norm_** **RMSE_abs_**			0.0257 -	0.0548 0.1297 *	0.0146 0.0463
**ramp**					
***V*_max_cond_**	(mV)	69.6	67.0	69.2	69.6
**RMSE_norm_** **RMSE_abs_**			0.1098 -	0.0396 0.0317 *	0.0262 0.0364

* Ion channel numbers *N_c_* used for simulation of the macroscopic current with the HMM model and calculation of RMSE_abs_ values were for activation N_c_act_ = 3088, deactivation N_c_deact_ = 2588, inactivation N_c_inact_ = 2588, and ramp N_c_ramp_ = 2388.

**Table 4:** Qualitative comparison of the HH, HMM and STB approaches.

	HH	HMM	STB
explainability of channel gating	+	+++	n.a.
flexibility and adaptability	+	+++	+
model complexity	+	+++	+
model accuracy	(<<) +	++ (>>)	+++
comp. burden optimization	++	+++	+
comp. burden simulation	+	++	+
experimental data for model parameterization	+++	+++ (>>)	+

Assessment of methods: n.a.: not represented by the model approach; low (+) to high (+++) scores; (<<) tends to be lower; (>>) tends to be higher.

**Figure A3:**
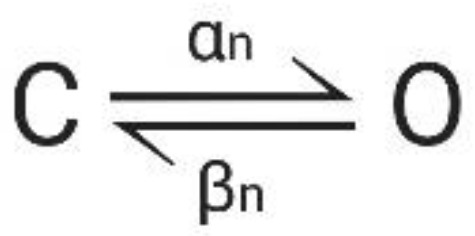
Transition reaction. C: closed state, O: open state, α: voltage-dependent forward transition, β: voltage-dependent backward transition.

## Data Availability

Experimental data, source code of the Kv1.1 HMM model parameterization and simulation, and visualized simulation results of all three models are available online at https://www.tugraz.at/en/institutes/hce/research-working-groups/research-data/kv1-1-model/ (accessed on 25 November 2021).

## Appendix A. Computational Modeling and Parameterization

The different modeling approaches were implemented in the simulation environment MATLAB (R2019b, MathWorks Inc.).

**HH model.** For the HH model, differential equations of activation and inactivation gates were solved numerically by the Forward Euler method according to Ranjan et al. [15], using a step size of ∆t = 1.10^−4^ s.

**HMM model.** The parametrization of the HMM was based on the averaged activation (n = 60) and deactivation (*n* = 37) data by a particle swarm optimization (PSO) algorithm (swarm size: 600; number of iterations: 10,000; function tolerance: 1 × 10^−6^) using the Global Optimization Toolbox (MathWorks Inc.). Defining $P_{S_{i},k}$ as the fraction of channels in a specific state *S_i_* at time-step *k,* the time evolution of the system could be described by the following set of autonomous difference equations:

$$\left[\begin{array}{c} P_{C_{1},k+1}\\ P_{C_{2},k+1}\\ P_{C_{3},k+1}\\ P_{C_{4},k+1}\\ P_{O,k+1}\\ P_{{IC}_{1},k+1}\\ P_{{IC}_{2},k+1}\\ P_{IN,k+1} \end{array}\right]=\left[\begin{array}{ccccccccc} 1-3\alpha \cdot dt & \beta \cdot dt & 0 & 0 & 0 & 0 & 0 & 0\\ 3\alpha \cdot dt & 1-(2\alpha +\beta )\cdot dt & 2\beta \cdot dt & 0 & 0 & 0 & 0 & 0\\ 0 & 2\alpha \cdot dt & 1-(\alpha +2\beta )\cdot dt & 3\beta \cdot dt & 0 & 0 & 0 & 0\\ 0 & 0 & \alpha \cdot dt & 1-(c+3\beta +x)\cdot dt & d\cdot dt & y\cdot dt & 0 & 0\\ 0 & 0 & 0 & c\cdot dt & 1-(d+\lambda +2x)\cdot dt & 0 & y\cdot dt & \eta \cdot dt\\ 0 & 0 & 0 & x\cdot dt & 0 & 1-(y+\sigma )\cdot dt & \varepsilon \cdot dt & 0\\ 0 & 0 & 0 & 0 & 2x\cdot dt & \sigma \cdot dt & 1-(y+\varepsilon +k)\cdot dt & m\cdot dt\\ 0 & 0 & 0 & 0 & \lambda \cdot dt & 0 & k\cdot dt & 1-(\eta -m)\cdot dt & m\cdot dt \end{array}\right]\cdot \left[\begin{array}{c} P_{C_{1},k}\\ P_{C_{2},k}\\ P_{C_{3},k}\\ P_{C_{4},k}\\ P_{O,k}\\ P_{{\mathrm{IC}}_{1},k}\\ P_{{\mathrm{IC}}_{2},k}\\ P_{IN,k} \end{array}\right]$$

The system was simulated with the MATLAB *lsim* function (MathWorks Inc.) over the entire simulation protocol, with a step size of dt = 5 × 10^−7^. The output vector was defined as $c^{T}=\left[\begin{array}{cccccccc} 0 & 0 & 0 & 0 & 1 & 0 & 0 & 0 \end{array}\right]$, to obtain the fraction of channels in the open state $P_{O,k}$ for each time-step k. The optimization defined the best choice for the voltage-dependent forward (α, λ, σ) and backward state transition rates (β, η, ε) and the constant state transition rates c, d, m, k, x, and y as well as the number of ion channels ($N_{C_{Kv1.1}}$) by fitting the resulting macroscopic ($I_{\mathrm{model}})$ current to the measured whole-cell current ($I_{\mathrm{data}})$:

(A1) $$\mathrm{RMSE}=\sum {\left(I_{\mathrm{model},k}-I_{\mathrm{data},k}\right)}^{2}$$

with $I_{\mathrm{model},k}=N_{C_{Kv1.1}}\cdot P_{O,k}\cdot g_{Kv1.1}\cdot \left(V-E_{K}\right)$.

Measured activation curves between 10.4 ms and 601.7 ms were considered for parameterization. For the deactivation curves, again, only the test pulses starting from 401.2 ms to 598.2 ms were used, excluding the depolarization pulse. In addition, to account for the lower number of voltage-levels of the deactivation protocol used, the deactivation curves were weighted for adequate consideration by a factor of 3.5.

To stochastically model the opening and closing of the single Kv1.1 ion channels for model validation, the *hmmgenerate* function (MathWorks Inc.) was used to generate a random sequence of states. The transition probability matrix T was defined as follows:

$$T=\left[\begin{array}{ccccccccc} & C_{1} & C_{2} & C_{3} & C_{4} & O & {\mathrm{IC}}_{1} & {\mathrm{IC}}_{2} & IN\\ C_{1} & 1-3\alpha \cdot dt & 3\alpha \cdot dt & 0 & 0 & 0 & 0 & 0 & 0\\ C_{2} & \beta \cdot dt & 1-\left(2\alpha +\beta \right)\cdot dt & 2\alpha \cdot dt & 0 & 0 & 0 & 0 & 0\\ C_{3} & 0 & 2\beta \cdot dt & 1-(\alpha +2\beta )\cdot dt & \alpha \cdot dt & 0 & 0 & 0 & 0\\ C_{4} & 0 & 0 & 3\beta \cdot dt & 1-(3\beta +c+x)\cdot dt & c\cdot dt & x\cdot dt & 0 & 0\\ O_{1} & 0 & 0 & 0 & d\cdot dt & 1-(d+2x+\lambda )\cdot dt & 0 & 2x\cdot dt & \lambda \cdot dt\\ IC1 & 0 & 0 & 0 & y\cdot dt & 0 & 1-(y+\sigma )\cdot dt & \sigma \cdot dt & 0\\ IC2 & 0 & 0 & 0 & 0 & y\cdot dt & \varepsilon \cdot dt & 1-(\varepsilon +y+k)\cdot dt & k\cdot dt\\ IN & 0 & 0 & 0 & 0 & \eta \cdot dt & 0 & m\cdot dt & 1-(\eta +m)\cdot dt \end{array}\right]$$

Occupancy was summarized for closed, open, and inactivated states at each time step for corresponding fractional occupancy plots.

**STB model.** The main advantage of this approach is that the model equations (see Equation (7)) do not have to be solved numerically. Instead, the identified model can be exported into the workspace of MATLAB where the obtained model can be further analyzed, linearized, or inserted into Simulink for a further application and simulation. The dynamic behavior of the ion channel is finally characterized by the transfer function and input/output nonlinearities.

In detail, we used the MATLAB Control System toolbox and the System Identification App. Note that a general system identification methodology contains key elements such as the experiment design, experiment, data preprocessing, fitting model to data, model validation, and audit [42].

For the STB model development, we assumed that a ramp stimulus protocol was a proper stimulus for the system identification. Using the System Identification App, we imported the time domain data and performed some data operations, including filtering, removing means, or transforming the data. Finally, we were able to define a mathematical model of the system represented as a nonlinear polynomial transfer function in state space. After analyzing different model concepts, we decided to use a nonlinear HW model because this model best fit the experimental data. After the model was validated, it could be exported to the MATLAB workspace and inserted into Simulink.

It should be noted that the HW model obtained with the System Identification App was represented in discrete—Z-domain—, which we then converted to the continuous domain since the opening/closing of ion channels is a continuous-time process. Finally, the HW model developed in MATLAB was fully functional for a further analysis, synthesis, and simulations of the Kv1.1 dynamics.

## Appendix B. Simulation of AP and Recovery Voltage Protocols with the HMM and STB Models

**AP:** Action potential protocol consisted of a 1.8 s long regular spiking action potential at a 30 mV voltage and frequency of 18 Hz, mimicking the physiological stimuli of pyramidal neurons.

**Recovery:** Recovery characteristics from inactivation were measured with 16 recovery pulses of a 200 ms duration at 50 mV after channel inactivation by 1.5 s long pre-pulses at 50 mV, and the recovery of the cells by holding them at −80 mV for variable times between 50 ms and 3.2 s in steps of 150 ms.

## Appendix D. Original Hodgkin–Huxley Formalism of the Potassium Current

The ionic current of an ion channel is given by the conductance *g*_x_ and driving force (*V* − *E*_x_) of the ion:

(A2) $$I_{x}=g_{x}\left(V-E_{x}\right)$$

with $g_{x}=\bar{g_{x}}y^{z}$.

In the Hodgkin–Huxley formalism, the macroscopic ion conductance *g*_x_ is described by various gates, controlling the flow of ions through the membrane. Each gate contains several independent gating particles z, which change between the open and closed positions, depending on the membrane potential. The gating variable y represents the probability of a single gating particle being in the open state. For several independent gating particles z, the probability of the entire gate being open, is given by *y*^z^.

The movement of gating particles between the closed and open state can be expressed as a reversible reaction with forward and backward rates *α*(V) and *β*(V):

Assuming a large number of ion channels, the probability y of individual gating particles being in the open state can be interpreted as the fraction of gating particles in the open position. Correspondingly, the fraction of gating particles in the closed state is 1 − *y*. Thus, the time evolution of the gating variable *y* can be described by a first-order differential equation:

(A3) $$\frac{dy}{dt}=\alpha_{y}\left(1-y\right)-\beta_{y}y$$

or

(A4) $$\frac{dy}{dt}=\frac{y_{\infty }-y}{\tau_{y}}$$

The general form of the time evolution for *y*(*t*) to a voltage step is:

(A5) $$y\left(t\right)=y_{\infty }\left(V\right)-\left(y_{\infty }\left(V\right)-y_{0}\right)\cdot e^{\frac{-t}{\tau_{y}\left(V\right)}}$$

where *y*_0_ is the starting point at time zero, $y_{\infty }$ the steady state value, and $\tau_{y}$ the time constant. Both, $y_{\infty }$ and $\tau_{y}$ are related to the voltage dependent rate coefficients *α*(*V*) and *β*(*V*), which can further be modeled by fitting empirical functions of the membrane potential to experimental data:

(A6) $$y_{\infty }=\frac{\alpha_{y}}{\alpha_{y}+\beta_{y}}$$

(A7) $$\tau_{y}=\frac{1}{\alpha_{y}+\beta_{y}}$$

As proposed by Hodgkin and Huxley [42], the best fit for the non-linear potassium conductance in the squid giant axon was achieved by assuming four independent gating particles for the activation gate *n*, leading to the following model equations for the potassium current *I**_K_*:

(A8) $$I_{K}=\bar{g_{K}}n^{4}\left(V-E_{K}\right)$$

(A9) $$\frac{dn}{dt}=\alpha_{n}\left(1-n\right)-\beta_{n}n$$

with $\alpha_{n}=0.01\frac{V+55}{1-exp^{\frac{-\left(V+55\right)}{10}}}$ and $\beta_{n}=0.125\cdot exp^{\frac{-\left(V+65\right)}{80}}.$
